# Retrorectal hamartoma: A ‘tail’ of two cysts!

**DOI:** 10.4103/0971-3026.63049

**Published:** 2010-05

**Authors:** Prasant Peter, Uttam George, Mark Peacock

**Affiliations:** Department of Radiodiagnosis, Christian Medical College and Hospital, Ludhiana, Punjab, India; 1 Department of Colorectal and General Surgery, Christian Medical College and Hospital, Ludhiana, Punjab, India

**Keywords:** Perirectal, retrorectal hamartoma, tailgut cysts

## Abstract

Retrorectal hamartomas or tail gut cysts are rare congenital anomalies most commonly seen in a retrorectal location; most common in middle aged women. This article describes the radiological appearance in two cases of tail gut cysts in males, one a child with a visible perianal swelling since birth and the other, a 72-year-old man with symptoms for one week. In both, the tailgut cysts were in a right perirectal location. Presentation in such a location in males, at extremes of age, is unusual for tailgut cysts.

## Introduction

Retrorectal hamartomas or tailgut cysts are rare congenital anomalies arising from the vestiges of the embryonic hindgut. They are more common in middle-aged women[1] and usually present as cysts in the retrorectal space.[2] We report the CT scan and MRI appearances, respectively, of two cases with tailgut cysts - one presenting shortly after birth and the other manifesting at 72 years, both in a right perirectal location. This presentation at the extremes of age in male subjects is unusual for tailgut cysts.

## Case 1

**Figure 1 (A, B):**
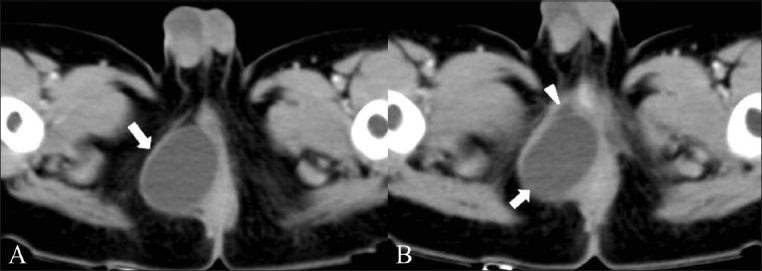
Axial CT scans show the tailgut cyst as a round, sharply-marginated lesion (arrow) with fluid contents, displacing the anal canal to the left, with a smaller peripheral cyst (arrowhead in B) seen anterior and inferior to the larger cyst

A 19-month-old male child presented to the pediatric outpatient department for evaluation of a large swelling in the perianal region, which was present since birth and had gradually increased in size. An earlier attempt at aspiration at one year of age revealed that it contained brownish fluid. An initial reduction in size of the mass following the aspiration was followed by gradual increase. On examination, the mass was compressible and nontender. CT scan showed a homogenous, well-defined, cystic lesion in the right perirectal/perianal region, displacing the rectum to the left [Figure 1A]. No solid component, calcification, or postcontrast enhancement was noted in the mass. A small peripheral cyst was also noted [Figure 1B]. No obvious communication with the adjacent bowel was noted.

The mass was excised *in toto*. Histopathology revealed a cystic lesion lined by cuboidal and columnar epithelium supported by fibroconnective tissue and skeletal muscle fibers, consistent with a cystic hamartoma.

## Case 2

**Figure 2 (A, B):**
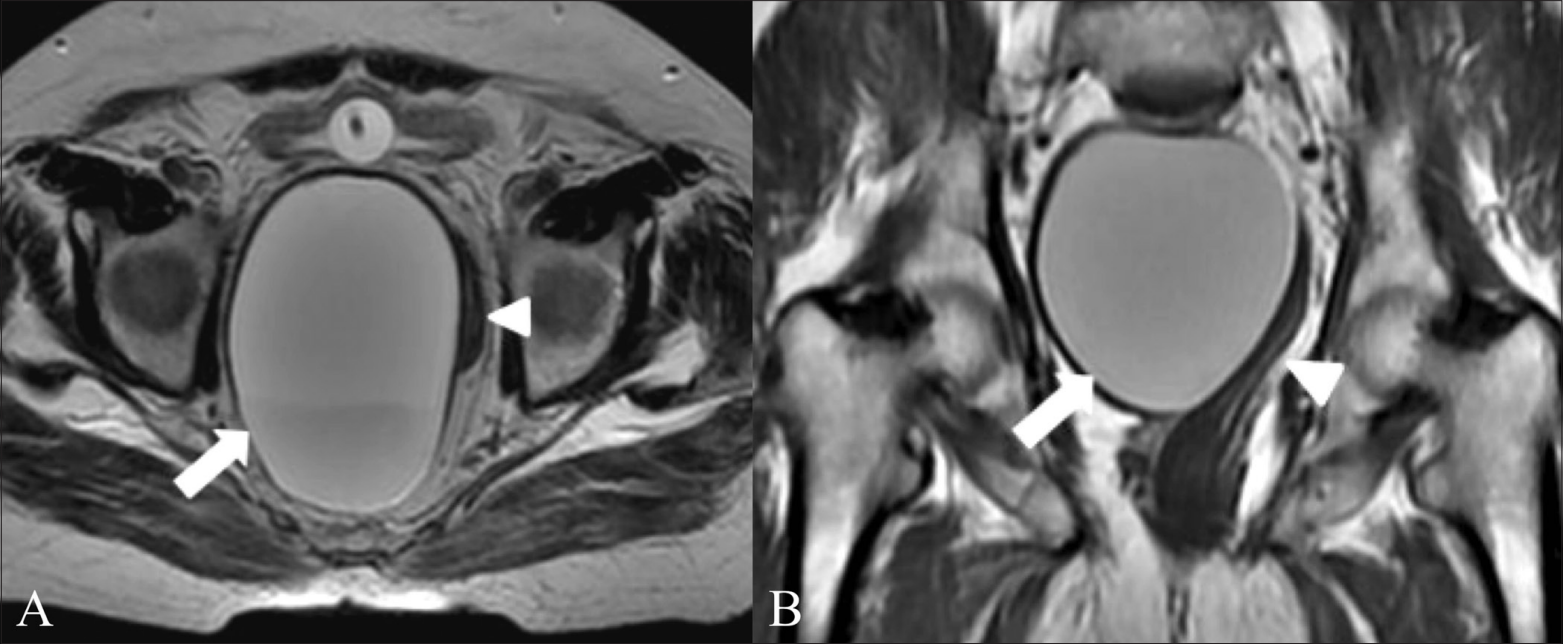
Axial T2W (A) and coronal T1W (B) MRI images show a large, hyperintense tailgut cyst (arrow) along the right margin of the rectum (arrowhead), which is displaced to the left

A 72-year-old male presented with urinary incontinence for a week. On rectal examination, there was mild tenderness and evidence of extrinsic compression of the rectum and anal canal from the 7 o'clock to 12 o'clock positions, without any mucosal involvement. Pelvic MRI showed a large ovoid, well-defined cystic lesion in close apposition to the right rectal wall, displacing the rectum to the left. Intralesional hyperintensity on both T1- and T2-weighted images, with layering suggestive of high protein or hemorrhagic contents was noted [Figure 2]. The patient underwent laparotomy and excision of the pelvic cyst. Intraoperatively, a 10 × 15 cm globular cystic mass was seen in the right perirectal region, containing about 800 ml of a dark greenish fluid. The cyst was excised and the patient had an uneventful recovery. Histopathology showed dense acute and chronic inflammatory infiltrate, with extensive hemorrhages and hemosiderin-laden macrophages and occasional epithelioid and foreign body-type giant cells; the findings were suggestive of an infected tailgut cyst.

## Discussion

Tailgut cysts are rare developmental cysts which, along with duplication cysts, are categorized under enteric cysts.[1] The tailgut is the distal-most portion of the hindgut and lies beyond the future anus. The anus develops by around 56 days and following this the tailgut undergoes involution. Failure of regression results in persistent vestiges, which may form into tailgut cysts.[3] The most common location is the presacral retrorectal space, with anterior rectal displacement.[2,4] The tailgut cyst in our first case appeared to be in a right perirectal location, displacing the rectum and anal canal to the left. In our second case, the cyst was seen to extend from a retrorectal location to the right of the rectum, again with left rectal displacement.

Presentation is usually as an asymptomatic retrorectal mass or with abdominal pain or constipation. Some patients may present with recurrent retrorectal abscesses or following multiple procedures for anal fistulae. On gross appearance they are unilocular or multilocular cystic lesions with mucoid contents. Microscopy shows a variety of epithelia without the presence of villi and crypts. An incomplete muscle layer is often associated with the cyst wall. Infected cysts show cyst wall fibrosis and breakdown of the epithelial lining. Rarely, malignant transformation may occur in the cyst.[5]

Assessment with USG is of limited value, showing only a multilocular cyst with debris and gelatinous material seen as internal echoes. On CT scan, tailgut cysts appear as a discrete, well-marginated mass of water or soft-tissue density as seen in our first patient. Calcification of the thin wall may rarely be seen.[1] In case of infection or, rarely, malignant transformation, loss of discrete margins and involvement of adjacent structures may be seen.[2] While histopathology suggested infection of the cyst in our second case, no obvious findings were appreciated on imaging.

A contrast study may have helped clarify the possibility of infection within the cyst. Though typically hypo intense on T1W and hyper intense on T2W images, a heterogeneous appearance on MRI may be seen owing to mucin, proteinaceous material, or hemorrhage within the cyst.[6,7] T1 and T2 hyperintensity was noted in our second case, which could be attributed to the infection and the extensive hemorrhage found on histopathology. A multilocular appearance with internal septae on T2W images has been described by Kim *et al*, as being specific for tailgut cysts.[6] However, neither of our cases showed the presence of septae. The presence of a small peripheral cyst accompanying the larger cyst has also been described by Yang *et al*.[4] A similar finding was identified in the CT scan of our first case (the child). A mass effect in large lesions has been described, with displacement of the adjacent rectum, as was observed in both our patients.

Most previous reports on tailgut cysts have described them as being more common in middle-aged women;[1–6,8] their occurrence in infants is rare,[2,3,5] with one report of in a two-year-old girl.[9] Both of our cases, however, occurred in males, one at each extreme of age, thus showing that tailgut cysts may occur over a wide age range. Also, unlike the common retrorectal location highlighted in previous reports, a predominantly right perirectal location was observed in both our patients.

We thus highlight the importance of considering tailgut cyst as a possible differential in any case of perirectal cyst, irrespective of age and sex, and the importance of imaging in assessing the characteristics and extent prior to surgery.
